# Clinical application value of metagenomic second-generation sequencing technology in hematologic diseases with and without transplantation

**DOI:** 10.3389/fcimb.2023.1135460

**Published:** 2023-06-16

**Authors:** Xia Zhang, Fang Wang, Jifeng Yu, Zhongxing Jiang

**Affiliations:** Department of Hematology, First Affiliated Hospital of Zhengzhou University, Zhengzhou, Henan, China

**Keywords:** hematological diseases, infection, pathogen, metagenomic next-generation sequencing, hematopoietic stem cell transplant

## Abstract

**Introduction:**

Hematological patients are at risk of infections. It is unknown whether the pathogenic microbial spectrum differs between HSCT and non-HSCT patients, and whether metagenomic next-generation sequencing (mNGS) of peripheral blood can be used as a substitute test specimen such as alveolar lavage.

**Methods:**

A retrospective study was conducted to evaluate the clinical application value of mNGS in hematological patients with and without HSCT.

**Results:**

Viruses were prevalent pathogens in both non-HSCT (44%) and HSCT (45%) patients, chiefly human cytomegalovirus and Epstein–Barr virus. In non-HSCT patients, Gram-negative bacilli accounted for 33% (predominantly Klebsiella pneumonia), and Gram-positive cocci accounted for 7% (predominantly Enterococcus faecium) of pathogens. However, in HSCT patients, Gram-negative bacilli accounted for 13% (predominantly Stenotrophomonas maltophilia), and Gram-positive cocci accounted for 24% (predominantly Streptococcus pneumonia) of pathogens. Mucor was the most common fungu s in two groups. The positive rate of pathogens by mNGS was 85.82%, higher than conventional detection (20.47%, P < 0.05). Mixed infection accounted for 67.00%, among which the mixed infection of bacteria and virus (25.99%) was the most common. 78 cases had pulmonary infection, the positive rate of traditional laboratory tests was 42.31% (33/78), and of mNGS in peripheral blood was 73.08% (57/78), showing a statistical difference (P = 0.000). The non-HSCT patients had a higher frequency of Klebsiella pneumonia (OR=0.777, 95% CI, 0.697-0.866, P = 0.01) and Torque teno virus (OR=0.883, 95% CI, 0.820-0.950, P = 0.031) infections than HSCT patients, while the rates of Streptococcus pneumonia (OR=12.828, 95% CI, 1.378-119.367, P = 0.016), Candida pseudosmooth (OR=1.100, 95% CI, 0.987-1.225, P = 0.016), human betaherpesvirus 6B (OR=6.345, 95% CI, 1.105-36.437, P = 0.039) and human polyomavirus 1 (OR=1.100, 95% CI, 0.987-1.225, P = 0.016) infections were lower. Leishmania could be detected by mNGS.

**Conclusion:**

mNGS of peripheral blood can be used as a substitute test method for hematological patients with pulmonary infection, the detection rate of mixed infections by mNGS was high, and mNGS has high clinical recognition rate and sensitivity in pathogen detection, and provides a basis for guiding the anti-infective treatment in hematological diseases with symptoms such as fever.

## Introduction

Patients with hematological diseases and recipients of hematopoietic stem cell transplantation (HSCT) are more prone to the development of infections due to immune reconstruction, the use of immunosuppressants, and other factors that cause damage to the immune system (Maschmeyer et al., 2019). Consequently, patients with hematopoietic malignancies are more vulnerable to infection caused by microbial organisms such as bacteria, viruses, and fungi due to immunocompromise. Infection is one of the main causes of death in patients with severe hematological diseases. In recent years, with the emergence of new pathogenic microorganisms, the increase in drug-resistant pathogenic microorganisms and the increase in immunosuppressed hosts, the morbidity and mortality of infection remain high, with a fatality rate among patients with sepsis reaching 50% (Chiu and Legrand, 2021). Severe acute bacterial infections are an enormous and growing health care burden, and they typically lack a timely diagnosis and thus adequate therapy in approximately 40% of clinical cases (Chao et al., 2020). In a study of 17, 990 patients with sepsis, Ricard Ferrer et al. (Ferrer et al., 2014) found that delayed antibiotic use led to sepsis and increased mortality. Invasive fungal infections are associated with unacceptably high mortality rates, killing approximately one and a half million people every year (Brown et al., 2012). Thus, it is of great importance to identify the pathogenic microorganisms as rapidly as possible in order to guide treatment. However, blood culture has a low sensitivity in probing many microorganisms, which can detect bacterial/fungal culture and the culture cycle is long (Hasanuzzaman et al., 2021). Quantitative real-time polymerase chain reaction (qRT–PCR) is quick and convenient, but it can only detect specific, suspected pathogens. Serum procalcitonin (PCT), C-reactive protein (CRP), erythrocyte sedimentation rate (ESR), (1,3)-β-D-glucan test (G test), and galactomannan test (GM test) have certain value in the diagnosis of infection. However, these indicators are affected by a variety of factors, and the specific pathogen cannot be identified (Cantey and Lee, 2021). There are also numerous pathogens that are difficult to detect, such as *Mycoplasma pneumoniae*, *Chlamydia pneumoniae*, *Cryptococcus neoformans*, *Pneumocystis carinii*, *Mycobacterium tuberculosis*, and common microorganisms. Despite these efforts, the etiology remains unknown in approximately 60% of cases, which results in delayed or ineffective treatments, increased mortality, and excessive health care costs (Schlaberg et al., 2017).

Metagenomic next-generation sequencing (mNGS) technology, as a diagnostic tool, is not susceptible to environmental interference, rapid, accurate and high positive rate, which can fulfill such a need. It overcomes the shortcomings of the traditional detection methods such as harsh culture conditions, long culture time and low positive rate. Through gene sequencing and database comparison, it can comprehensively analyze all possible pathogenic microorganisms in samples, with the characteristics of a short detection period and comprehensive detection (Gu et al., 2019).

It is also unknown whether the pathogenic microbial spectrum differs between HSCT and non-HSCT patients. Patients with hematologic diseases are also prone to concurrent thrombocytopenia and infection. Therefore, due to the risk of bleeding, the inability to obtain alveolar lavage solution through bronchoscopy presented great difficulties in the diagnosis and treatment of pathogenic microorganisms in patients with hematologic diseases. We will investigate whether mNGS of peripheral blood can be used as a substitute test specimen, when alveolar lavage is difficult to obtain. Consequently, we speculate that mNGS technology could improve clinical management methods of hematology patients with fever. This study retrospectively analyzed the pathogenic microorganism detection results and prognosis of hematological patients with fever from May 2021 to March 2022. It is expected to provide guidance for the treatment of patients and the prediction of the clinical prognosis.

## Methods

### Patient enrollment and clinical data collection

This retrospective study was approved by the Ethics Review Committee of the First Affiliated Hospital of Zhengzhou University, and written informed consent was obtained from all subjects or their guardians. Clinical samples of hospitalized patients were collected from May 2021 to March 2022. The inclusion criteria were as follows: patients with obvious symptoms of infection, including (1) hematologic diseases; (2) any of the following symptoms or signs: fever (>38 °C), coughing, expectoration, pleural effusion, seroperitoneum, urinary tract infections, diarrhea, cardiac effusion, deep abscess, chest CT showing lung infection, etc. The clinical data of the patients were recorded, and blood, urine, excrement, and sputum samples were collected depending on their clinical condition. The clinical samples were used to perform bacterial culture, qRT–PCR or deoxyribonucleic acid (DNA) extraction followed by high-throughput sequencing.

### Specimen collection

Clinical specimens, such as blood samples, bone marrow liquid, sputum, pus, nasopharyngeal/oropharyngeal/perianal swabs, pleural effusion, ascites, mid-stream urine samples, stool, pericardial effusion, and secretion, were obtained from patients as soon as possible after presentation and were collected before antimicrobial therapy began. Samples were stored at −80°C until transferred to the central laboratory.

### Pathogen detection

The samples were placed in anaerobic and aerobic culture bottles and immediately sent to the bacterial room of our hospital for pathogen culture. Epstein–Barr virus (EBV), human cytomegalovirus (CMV) and BK virus were detected by PCR in patients with virus infection. The samples were placed in EDTA tubes and special virus transfer tubes and sent to Genskey Co., Ltd., Beijing, for mNGS detection of pathogenic microorganisms (Jing et al., 2021), which is known as shotgun sequencing with the following advantages: high sequencing throughput, high sensitivity, early identification of pathogens, and detection of special pathogens.

### Sampling and mNGS sequencing

The metagenomic next-generation sequencing method was used as described in the reference (Jing et al., 2021; Zhang et al., 2021). Samples were collected by BCT tubes, centrifuged at 1600g for 10 min, and cfDNA was extracted from plasma supernatant. In the experiment, both internal control and external negative control were used in QC. DNA was extracted using the TIANamp Micro DNA Kit (TIANGEN, China) and quantified by fluorometry (ThermoFisher Scientific, USA) according to the manufacturer’s recommendation.

DNA library was constructed according to the protocol of the BGISEQ-200 sequencing platform by DNA fragmentation, end-repair, adapter-ligation, and PCR amplification, which was qualified by Agilent 2100 (Agilent Technologies, USA) and Qubit 4.0 (Thermo Fisher, USA). Qualified double-stranded DNA libraries became single-stranded DNA by denaturation and cyclization. By rolling circle amplification (RCA), single-stranded circular DNA was transformed into DNA nanoballs (DNBs). The DNBs were carried out quality control by Qubit 4.0. Qualified DNBs were loaded into the flow cell and sequenced (50 bp, single-end) on the BGISEQ-200 platform (Zhang et al., 2021). Up to 20 libraries per batch were pooled to be sequenced on Illumina NextSeq 550 sequencers using a 75-cycle single-end sequencing strategy.

### Bioinformatics pipeline

Adapters and low quality and short (<35bp) of reads were removed to obtain clean reads by using fastp software (Chen et al., 2018). Human host sequences were excluded by mapping to the human reference genome (hg38) by using STAR alignment (Dobin et al., 2013). Low complexity and duplicated reads were removed by using PRINSEQ algorithms (Schmieder and Edwards, 2011). The remaining clean reads were blasted against in-house microbial genome databases, which were mainly downloaded from NCBI (ftp://ftp.ncbi.nlm.nih.gov/genomes/) using Kraken2 software (Wood et al., 2019). In terms of species-specific read number, reads per million and genome coverage, the sequencing data list was analyzed.

### Statistical analysis

Data were analyzed using SPSS 22.0 software (SPSS, Chicago, IL, US). Quantitative data were compared between the two groups using a t-test (for a normal distribution) or a nonparametric test (Mann–Whitney Test, not a normal distribution). For comparisons of the categorical data, the chi-squared (χ2) test was used. Data analysis was performed using GraphPad Prism 9 software (GraphPad Software, La Jolla, California). P < 0.05 was considered statistically significant.

## Results

### Patient characteristics and clinical samples

The characteristics of the 103 patients are summarized in **Table 1**. The median age was 39.3 years old, with 72 male and 31 female patients. There were 28 patients underwent HSCT and 75 patients with hematologic diseases who did not undergo HSCT. The underlying diseases included acute myeloid leukemia (n=34), acute lymphoblastic leukemia (n=12), acute mixed lineage leukemia (n=2), myelodysplastic syndromes (n=10), malignant lymphoma (n=5), chronic myeloid leukemia (n=2), chronic lymphocytic leukemia (n=2), multiple myeloma (n=7), hemophagocytic lymphohistiocytosis (n=5), aplastic anemia (n=8) and others (inclduing paroxysmal nocturnal hemoglobinuria, Henoch-Schonlein purpura, immune thrombocytopenia, and fever of undetermined origin (FUO), (n= 16). mNGS was performed 127 person-times. Clinical samples revealed 109 cases with clear pathogens and 18 with no pathogens. We analyzed the samples of pulmonary infection, 78 cases had pulmonary infection, the positive rate of traditional laboratory tests such as sputum culture and blood culture was 42.31% (33/78), and the positive rate of mNGS in peripheral blood was 73.08% (57/78), showing a statistical difference (P = 0.000).

**Table 1:** Patient characteristics.

Variables	cases	ratio (%)	P
Age, year, median (range)	39.3 (14~67)		
Gender (male/female)	72/31		
Underlying disease			
Leukemia			
acute myeloid leukemia	34	33.01	
acute lymphoblastic leukemia	12	11.65	
acute mixed lineage leukemia	2	1.94	
chronic myeloid leukemia	2	1.94	
chronic lymphocytic leukemia	2	1.94	
Myelodysplastic syndromes	10	9.71	
Malignant lymphoma	5	4.85	
Multiple myeloma	7	6.80	
Hemophagocytic lymphohistiocytosis	5	4.85	
Aplastic anemia	8	7.77	
Others (ITP, etc.)	16	15.53	
Status at transplant			
HSCT	28	27.18	
Non-HSCT	75	72.82	
Pathogens			
clear pathogens	109	85.83	
no pathogens	18	14.17	
Pulmonary infection	78		0.000
conventional testing		42.31	
mNGS		73.08	

ITP, immune thrombocytopenia; allo-HSCT, allogeneic hematopoietic stem cell transplantation; auto-HSCT, autologous hematopoietic stem cell transplantation.

### Clinical assessment

The results of mNGS were assessed clinically by physicians. To determine whether the pathogenic microorganism was the pathogen causing the patient’s symptoms, the physicians evaluated the results in combination with the patient’s clinical symptoms and other examinations, such as PCT, CRP, ESR, G test and GM test, blood culture, features of lung imaging, and fibreoptic bronchoscopy. It was considered a component of the etiology if the index patient met the clinical criteria of infection, whether confirmed by conventional tests or not.

Treatment regimens were adjusted according to the results of pathogenic microorganisms. Escalation of the antibiotics avoided empirical combinations, while de-escalation, with initial broad-spectrum antibiotic combinations, could minimize the collateral damage associated with antibiotic overuse. However, based on the clinical manifestations and test results, some results of the mNGS were considered meaningless, and the original treatment was continued (**Table 2**). Antifungal prophylaxis should not be used routinely in all patients with neutropenia. The rationale for antifungal prophylaxis is to prevent fungal infections in a specific group of high-risk patients, especially those with longer durations of neutropenia or with GVHD after allogeneic HSCT. Antifungal agents include azoles, amphotericin B products, and echinocandins and its selection is determined by the disease and therapy strategy. Therefore, the physician’s clinical judgment is pivotal in the evaluation and modification in the antimicrobial regimen.

**Table 2:** Direction of clinical treatment by mNGS.

Clinical benefit of mNGS	mNGS guidance for patient management
Initiation therapy of antimicrobial agents	mNGS detected pathogens and directed application of antimicrobial agents.
De-escalation therapy of antimicrobial agents	According to the results of mNGS and traditional test, the pathogenic microorganisms were identified and the empirical anti-infective drugs were adjusted.
Upgradation therapy of antimicrobial agents	According to the results of mNGS and traditional test, the pathogenic microorganisms were identified and appropriate antimicrobial agents were selected.
Identification of pathogenic micro- organisms	mNGS detected pathogens and directed application of antibiotics.
No clinical significance	Combined with clinical symptoms and traditional test, it was ascertained that some pathogenic microorganisms had no clinical significance.

### The coverage of sequencing results in hematologic diseases with and without transplantation

**Figure 1:**
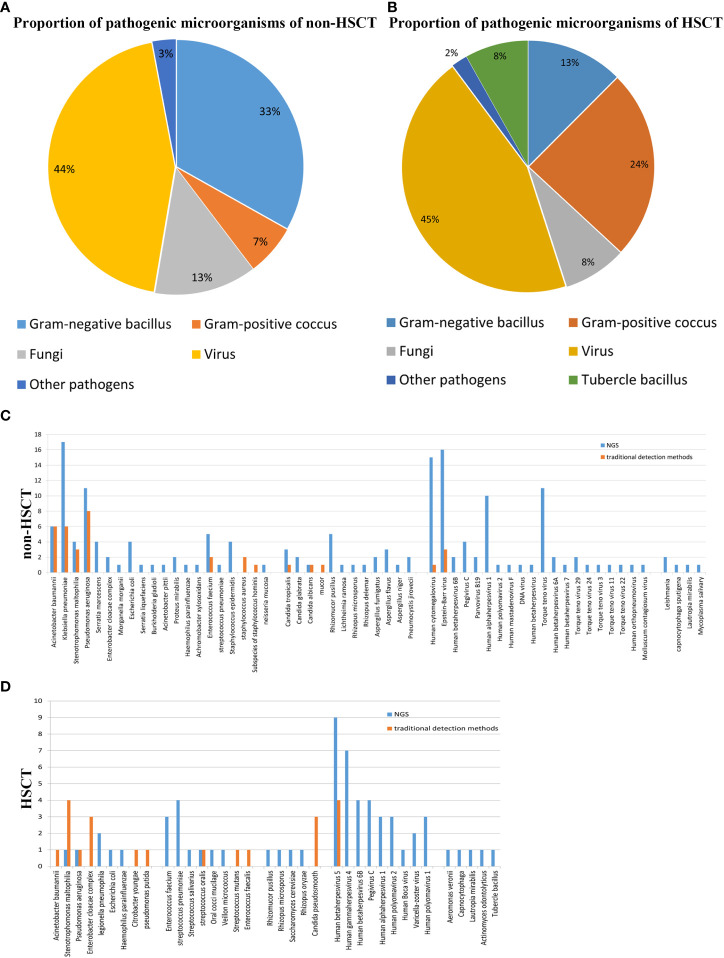
Patient characteristics and clinical samples. **(A)** The mNGS results revealed that bacteria occupied the bulk of pathogenic microorganisms in patients with hematological disease but not those with HSCT. Gram-negative bacillus accounted for a greater proportion of the bacteria. The proportion of virus was high in both the transplantation and nontransplantation groups. **(B)** In the nontransplantation group, *Klebsiella pneumonia*, *Pseudomonas aeruginosa* and *Acinetobacter baumannii* were the top 3 bacteria. However, *Stenotrophomonas maltophilia* and *Streptococcus pneumonia* were commonly found in the HSCT group. Mucor was the most common fungus, and human cytomegalovirus and Epstein–Barr virus were commonly found both in the transplantation and nontransplantation groups. **(C)** The detection rates of *Acinetobacter baumannii*, *Klebsiella pneumonia*, *Stenotrophomonas maltophilia*, *Pseudomonas aeruginosa*, *Enterococcus faecium* and *Staphylococcus aureus* in non-HSCT patients were higher than those of other bacteria. **(D)** EBV and CMV were the most common pathogenic microorganisms. Non-HSCT, non-hematopoietic stem cell transplant; HSCT, hematopoietic stem cell transplant.

The mNGS results from our study revealed that viruses were the predominant pathogenic microorganisms in both non-HSCT (44%) and HSCT (45%) patients. Gram-negative bacilli accounted for 33%, and Gram-positive cocci accounted for 7% of pathogens in non-HSCT patients. However, Gram-negative bacilli accounted for 13% and Gram-positive cocci accounted for 24% of pathogens in HSCT patients. The percentage of fungi in non-HSCT patients (13%) was higher than that in HSCT patients (8%). Tubercle bacillus (8%) was detected by mNGS in HSCT patients but not in non-HSCT patients. Other pathogens (3%), such as *Mycobacterium tuberculosis* and Leishmania, were detected in FUO patients (**Figures 1A, B**). In the non-HSCT group, *Klebsiella pneumonia*, *Pseudomonas aeruginosa* and *Acinetobacter baumannii* were the top 3 bacteria. Whereas, *Stenotrophomonas maltophilia*, *Streptococcus pneumonia*, *Enterobacter cloacae* complex and *Enterococcus faecium* were bacteria that were commonly found in the HSCT group. In both the non-HSCT and HSCT patient groups, Mucor was the most common fungus, and CMV and EBV were commonly found. The detection rate by mNGS method was higher than traditional detecting methods (**Figures 1C, D**).

### Higher detection rate of pathogens by mNGS

**Figure 2:**
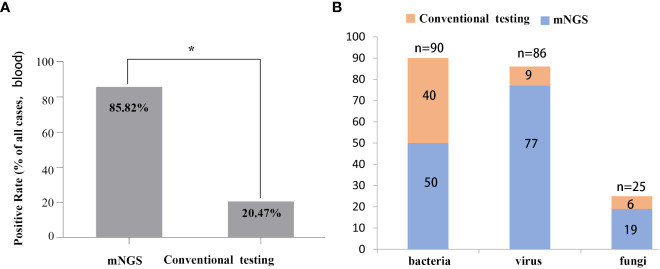
Comparison between mNGS and conventional testing for detecting pathogens in clinical samples. **(A)** Differences in rates of positivity by cases, *P* < 0.05. **(B)** Differences in the number of cases for pathogens detected by mNGS and the conventional method.

The positive rates of pathogens in clinical samples detected by mNGS and conventional testing were compared. The positive rate of pathogenic microorganisms detected by mNGS was 85.82%, which was higher than conventional detection (20.47%, P < 0.05) (**Figure 2A**). The number of cases using mNGS and traditional methods to detect pathogens were quite different. Fifty cases of bacteria were detected by mNGS, and 40 cases were detected by traditional methods. Seventy-seven cases of viruses were detected by mNGS, 9 cases were detected by the traditional method, 19 cases of fungi were detected by mNGS, and 6 cases were detected by the traditional method (**Figure 2B**).

### Spectra of pathogens and consistency of detection revealed by mNGS and conventional testing in hematological patients with and without transplantation

**Figure 3:**
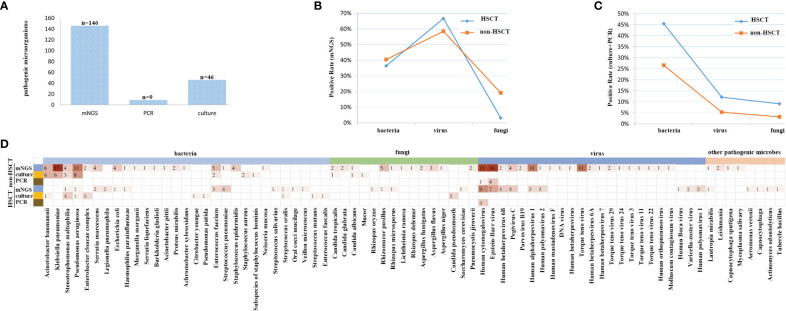
The coverage of sequencing results, focusing on all samples, and its high sensitivity for special pathogens. **(A)** The detection rate of mNGS was the highest among the three methods. **(B)** The detection rate of viruses was higher by mNGS, and after standardization of the rate, there were significant differences in bacteria, fungi and viruses between non-HSCT and HSCT patients, *P* < 0.05. **(C)** The detection rate of bacteria was the highest, and the detection rate of fungi was the lowest. There were no significant differences in bacteria, fungi and viruses between non-HSCT and HSCT patients, *P* > 0.05. **(D)** The infection spectrum indicated that the detection rate of pathogenic microorganisms was higher in patients without HSCT.

mNGS and conventional testing were performed in a total of 127 clinical samples. In the HSCT patient group, there were 12 cases of bacteria, 22 cases of viruses, 1 case of fungus detected by mNGS, 15 cases of bacteria, 4 cases of viruses, and 3 cases of fungi detected by conventional testing, such as PCR and/or culture. In the non-HSCT hematology patient group, there were 38 cases of bacteria, 55 cases of viruses, 18 cases of fungi detected by mNGS and 25 cases of bacteria, 5 cases of viruses, and 3 cases of fungi detected by conventional testing, such as PCR and/or culture. A total of 146 potential pathogens were identified by mNGS; 9 cases of viruses were detected by PCR, and 46 cases were detected by culture. The detection positive rate of mNGS was the highest among the three detection methods (**Figure 3A**).

The positive rate of viruses detected by mNGS was higher than that of traditional detection methods (66.7% *vs*. 12.12%), while for bacteria it was 36.6% *vs*. 45.45%, and for fungi it was 3.03% *vs*. 9.09% in the HSCT patient group. In the non-HSCT patient group, the positive rate of viruses detected by mNGS (58.51%) was higher than that of traditional detection methods (5.32%), for bacteria it was 40.43% *vs*. 26.59%, and for fungi it was 19.15% *vs*. 3.19%. The pathogens identified by mNGS and conventional testing were cross-validated by using either another assay or other samples collected from the same patient. There were significant differences in bacteria, fungi and viruses between non-HSCT and HSCT patients (*P* < 0.05) (**Figures 3B, C**). Among bacterium, fungus and virus groups, the detection rate of bacteria by culture was the highest while the detection rate of fungi was the lowest. There were no significant differences in bacteria, fungi and viruses detection rates between non-HSCT and HSCT patients (*P* > 0.05) (**Figure 3C**). The infection spectrum indicated that the detection rate of pathogenic microorganisms was higher in patients without hematopoietic stem cell transplantation (**Figure 3D**).

Consistency of detection was revealed by mNGS and conventional testings in hematological patients. Samples of 44 patients underwent mNGS, PCR, G and GM tests and culture testings simultaneously. EBV and/or CMV were detected as positive by mNGS but negative by PCR in 9 contemporaneous samples of the patients. However, the detection results were consistent in 35 of the patients. The consistency ratio between mNGS and traditional detection methods was 79.55%. Pathogens were identified in 28 patients by mNGS in combination with another laboratory testing (**Table 3**).

**Table 3:** Pathogens identified by mNGS in combination with other laboratory testing.

Number	Diagnosis	Type of clinical sample	Pathogenic microorganism	Validation method
2	AML	blood, sputum	Candida tropicalis	culture, G test
14	AML	blood, sputum, perianal swab	Rhizomucor pusillus	G test
15	AML	blood, sputum, perianal swab	Rhizomucor pusillus	G test
16	AML	blood, stool	Rhizomucor pusillus	G test
36	MM	blood,sputum,mid-stream urine	Candida albicans	culture, G test
38	AMLL	blood	Klebsiella pneumoniae	culture
40	ALL	blood,secretion	Pseudomonas aeruginosa	culture
41	AML	blood,secretion	Pseudomonas aeruginosa	culture
43	AML	blood,sputum,perianal swab	Klebsiella pneumoniae	culture
47	MDS	blood	Candida tropicalis	G test
48	HLH	blood, mid-stream urine, oropharyngeal swab	EBV	PCR
53	HLH	blood	EBV	PCR
54	AA	blood	Lichtheimia ramosa	G test, mNGS dynamic monitoring
58	AML	blood, sputum, mid-stream urine	Pseudomonas aeruginosa	culture
70	Other	blood,pleural effusion	EBV	PCR
77	AML	blood,stool	Klebsiella pneumoniae	culture
79	ALL	blood	Pseudomonas aeruginosa	culture
82	AML	blood	CMV	PCR
84	HLH	blood,sputum,pleural effusion	Klebsiella pneumoniae	culture
88	MDS	blood,sputum	Pseudomonas aeruginosa	culture
94	MDS	blood	Pseudomonas aeruginosa	culture
96	AMLL, HSCT	blood, perianal swab	Escherichia coli	culture
98	PNH, HSCT	blood, perianal swab	CMV	PCR
104	MDS, HSCT	blood,stool	Stenotrophomonas maltophilia	culture
107	AA, HSCT	blood, perianal swab	EBV, CMV	PCR
113	AA, HSCT	blood, perianal swab	EBV, CMV	PCR
117	MDS, HSCT	blood, perianal swab	Rhizomucor pusillus	G test
118	AA, HSCT	blood	CMV	PCR

AML, acute myeloid leukemia; ALL, acute lymphoblastic leukemia; MM, multiple myeloma; AMLL, acute mixed lineage leukemia; MDS, myelodysplastic syndromes; HLH, hemophagocytic lymphohistiocytosis; AA, aplastic anemia; PNH, paroxysmal nocturnal hemoglobinuria; HSCT, hematopoietic stem cell transplantation; EBV, Epstein-barr virus; CMV, Human cytomegalovirus; PCR, polymerase chain reaction; G test, (1,3)-β-D-glucan test.

### Different microbial etiologies in hematological patients with and without transplantation

**Figure 4:**
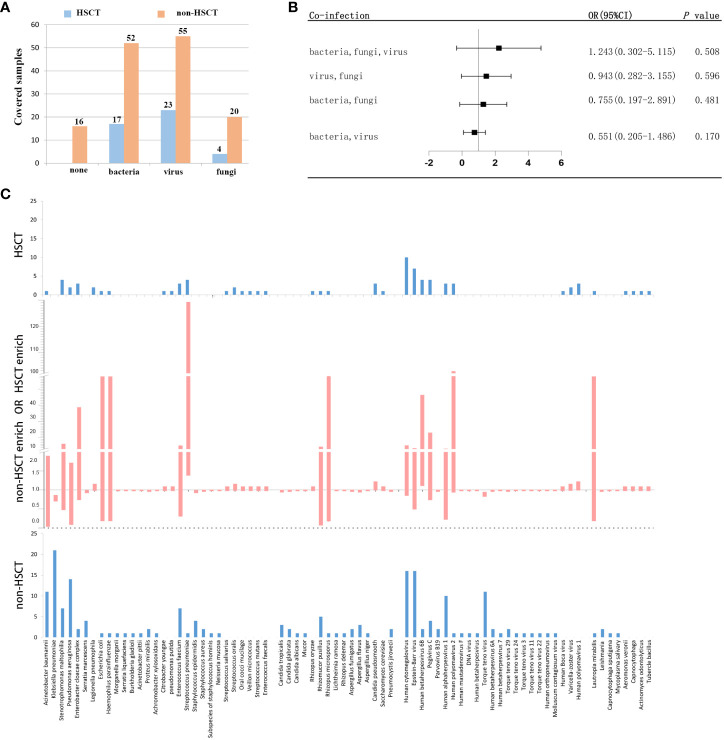
Different microbial etiologies in HSCT and non-HSCT patients. **(A)** Distribution of different types of microbial etiologies. **(B)** Potential coinfections in non-HSCT and HSCT patients. **(C)** Spectra of pathogenic microorganisms in HSCT and non-HSCT patients. The middle chart shows pathogenic microorganisms that were statistically significant (*P* < 0.05).

To further evaluate the microbial etiology of infection in hematological diseases, the potential pathogens between the HSCT and non-HSCT patient groups were compared. Combined with mNGS and conventional testing, pathogenic microorganisms in clinical samples were detected in all of the HSCT patients. There were no pathogens detected in 16 non-HSCT patients. Bacteria were detected in the HSCT group (17 cases) and non-HSCT group (52 cases), while viruses were detected in the HSCT group (23 cases) and non-HSCT group (55 cases), and fungi were detected in the HSCT group (4 cases) and non-HSCT group (20 cases) (**Figure 4A**). There were 3 patients infected by bacteria, viruses and fungi simultaneously in the HSCT patient group, whereas 7 patients in the non-HSCT group. The odd ratio (OR) (95% confidence interval, CI) between the non-HSCT patient group and HSCT group was 1.243 (0.302-5.115). Viruses and fungi coexisted in 12 non-HSCT patients and 4 HSCT patients. The OR (95% CI) between them was 0.943 (0.282-3.155). Both bacteria and fungi were present in 11 non-HSCT patients and 3 HSCT patients. The OR (95% CI) between them was 0.755 (0.197-2.891). Both bacteria and viruses were present in 27 non-HSCT patients and 6 HSCT patients. The OR (95% CI) between them was 0.551 (0.205-1.486). There were no significant differences between any of the above groups (*P*> 0.05). Patients with mixed infection accounted for 67.00%. Among them, the mixed infection of bacteria and virus (25.99%) was the most common (**Figure 4B**). However, we found statistically significant differences in pathogens between the non-HSCT patient group and HSCT group (**Figure 4C**). The non-HSCT patients had a higher frequency of *Klebsiella pneumonia* (OR=0.777, 95% CI, 0.697-0.866, *P* = 0.01) and *Torque teno* virus (OR=0.883, 95% CI, 0.820-0.950, *P* = 0.031) infections than HSCT patients, while the rates of *Streptococcus pneumonia* (OR=12.828, 95% CI, 1.378-119.367, *P* = 0.016), *Candida pseudosmooth* (OR=1.100, 95% CI, 0.987-1.225, *P* = 0.016), human betaherpesvirus 6B (OR=6.345, 95% CI, 1.105-36.437, *P* = 0.039) and human polyomavirus 1 (OR=1.100, 95% CI, 0.987-1.225, *P* = 0.016) infections were lower in non-HSCT patients (**Table 4**).

**Table 4:** Spectra of pathogenic organisms between non-HSCT and HSCT patients.

Pathogens	Organisms	non-HSCT (94)	HSCT (33)	*P* value	Odds ratio (95% CI)
Acinetobacter baumannii	bacterium	11	1	0.128	0.236 (0.029-1.901)
Klebsiella pneumoniae	bacterium	21	0	0.010	0.777 (0.697-0.866)
Stenotrophomonas maltophilia	bacterium	7	4	0.309	1.714 (0.468-6.280)
Pseudomonas aeruginosa	bacterium	14	2	0.156	0.369 (0.079-1.717)
Enterobacter cloacae complex	bacterium	2	3	0.110	4.600 (0.733-28.849)
Serratia marcescens	bacterium	4	0	0.295	0.957 (0.917-0.999)
Legionella pneumophila	bacterium	0	2	0.066	1.065 (0.976-1.161)
Escherichia coli	bacterium	1	1	0.454	2.906 (0.177-47.829)
Haemophilus parainfluenzae	bacterium	1	1	0.454	2.906 (0.177-47.829)
Morganella morganii	bacterium	1	0	0.740	0.989 (0.969-1.010)
Serratia liquefaciens	bacterium	1	0	0.740	0.989 (0.969-1.010)
Burkholderia gladioli	bacterium	1	0	0.740	0.989 (0.969-1.010)
Acinetobacter pittii	bacterium	1	0	0.740	0.989 (0.969-1.010)
Proteus mirabilis	bacterium	2	0	0.546	0.979 (0.950-1.008)
Achromobacter xylosoxidans	bacterium	1	0	0.740	0.989 (0.969-1.010)
Citrobacter youngae	bacterium	0	1	0.260	1.031 (0.971-1.095)
Pseudomonas putida	bacterium	0	1	0.260	1.031 (0.971-1.095)
Enterococcus faecium	bacterium	7	3	0.508	1.243 (0.302-5.115)
Streptococcus pneumoniae	bacterium	1	4	0.016	12.828 (1.378-119.367)
Staphylococcus epidermidis	bacterium	4	0	0.295	0.957 (0.917-0.999)
Staphylococcus aureus	bacterium	2	0	0.546	0.979 (0.950-1.008)
Subspecies of staphylococcus hominis	bacterium	1	0	0.740	0.989 (0.969-1.010)
Neisseria mucosa	bacterium	1	0	0.740	0.989 (0.969-1.010)
Streptococcus salivarius	bacterium	0	1	0.260	1.031 (0.971-1.095)
Streptococcus oralis	bacterium	0	2	0.066	1.065 (0.976-1.161)
Oral cocci mucilage	bacterium	0	1	0.260	1.031 (0.971-1.095)
Veillon micrococcus	bacterium	0	1	0.260	1.031 (0.971-1.095)
Streptococcus mutans	bacterium	0	1	0.260	1.031 (0.971-1.095)
Enterococcus faecalis	bacterium	0	1	0.260	1.031 (0.971-1.095)
Candida tropicalis	fungus	3	0	0.402	0.968 (0.933-1.004)
Candida glabrata	fungus	2	0	0.546	0.979 (0.950-1.008)
Candida albicans	fungus	1	0	0.740	0.989 (0.969-1.010)
Mucor	fungus	1	0	0.740	0.989 (0.969-1.010)
Rhizopus oryzae	fungus	0	1	0.260	1.031 (0.971-1.095)
Rhizomucor pusillus	fungus	5	1	0.508	0.556 (0.063-4.494)
Rhizopus microsporus	fungus	1	1	0.454	2.906 (0.177-47.829)
Lichtheimia ramosa	fungus	1	0	0.740	0.989 (0.969-1.010)
Rhizopus delemar	fungus	1	0	0.740	0.989 (0.969-1.010)
Aspergillus fumigatus	fungus	2	0	0.546	0.979 (0.950-1.008)
Aspergillus flavus	fungus	3	0	0.402	0.968 (0.933-1.004)
Aspergillus niger	fungus	1	0	0.740	0.989 (0.969-1.010)
Candida pseudosmooth	fungus	0	3	0.016	1.100 (0.987-1.225)
Saccharomyces cerevisiae	fungus	0	1	0.260	1.031 (0.971-1.095)
Pneumocystis jirovecii	fungus	2	0	0.546	0.979 (0.95-1.008)
Human cytomegalovirus	virus	16	10	0.087	2.12 (0.847-5.302)
Epstein-barr virus	virus	16	7	0.382	1.312 (0.486-3.543)
Human betaherpesvirus 6B	virus	2	4	0.039	6.345 (1.105-36.437)
Pegivirus C	virus	4	4	0.121	3.103 (0.730-13.200)
Parvovirus B19	virus	2	0	0.546	0.979 (0.950-1.008)
Human alphaherpesvirus 1	virus	10	3	0.550	0.840 (0.216-3.259)
Human polyomavirus 2	virus	1	3	0.054	9.300 (0.932-92.788)
Human mastadenovirus F	virus	1	0	0.740	0.989 (0.969-1.010)
DNA virus	virus	1	0	0.740	0.989 (0.969-1.010)
Human betaherpesvirus	virus	1	0	0.740	0.989 (0.969-1.010)
Torque teno virus	virus	11	0	0.031	0.883 (0.820-0.950)
Human betaherpesvirus 6A	virus	2	0	0.546	0.979 (0.950-1.008)
Human betaherpesvirus 7	virus	1	0	0.740	0.989 (0.969-1.010)
Torque teno virus 29	virus	2	0	0.546	0.979 (0.950-1.008)
Torque teno virus 24	virus	1	0	0.740	0.989 (0.969-1.010)
Torque teno virus 3	virus	1	0	0.740	0.989 (0.969-1.010)
Torque teno virus 11	virus	1	0	0.740	0.989 (0.969-1.010)
Torque teno virus 22	virus	1	0	0.740	0.989 (0.969-1.010)
Human orthopneumovirus	virus	1	0	0.740	0.989 (0.969-1.010)
Molluscum contagiosum virus	virus	1	0	0.740	0.989 (0.969-1.010)
Human Boca virus	virus	0	1	0.260	1.031 (0.971-1.095)
Varicella-zoster virus	virus	0	2	0.066	1.065 (0.976-1.161)
Human polyomavirus 1	virus	0	3	0.016	1.100 (0.987-1.225)
other	other	5	5	0.121	2.619 (0.742-9.240)

HSCT, hematopoietic stem cell transplant.

### Evaluation of the application of mNGS in hematological patients with and without transplantation in the real world

**Figure 5:**
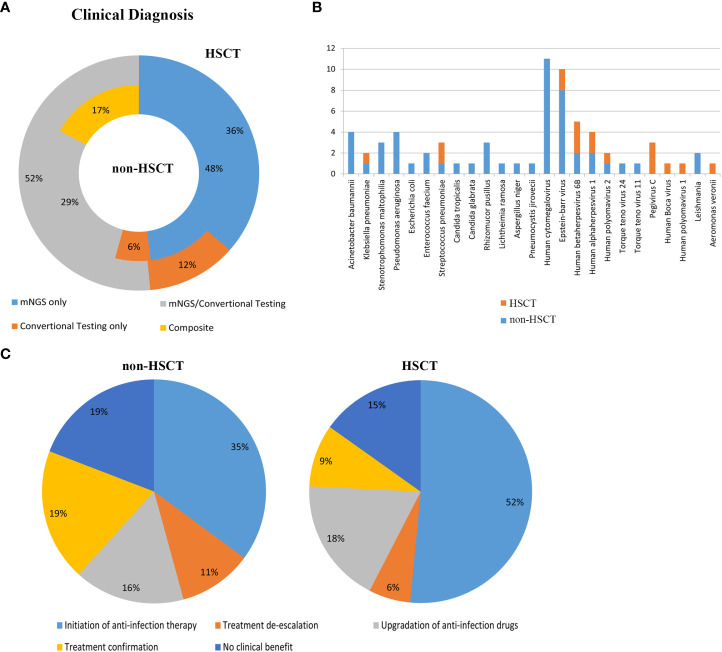
Evaluation of the application of mNGS in the real world. **(A)** Clinical diagnosis in the non-HSCT and HSCT groups. mNGS/conventional, pathogens identified by both methods; composite, pathogens identified by combining mNGS, the conventional testing results and clinical signs and symptoms; mNGS only and conventional testing only, pathogens identified by only a single method. Inner circle, non-HSCT; Outer circle, HSCT. **(B)** Clinically determined pathogens only detected by mNGS, not by conventional methods in hematological patients. **(C)** Evaluation of the clinical impact of mNGS in non-HSCT and HSCT patients.

Clinical samples from patients were tested by mNGS and conventional methods. The application of mNGS in the real world was evaluated. 45 cases (48%) of pathogens were detected by mNGS only in non-HSCT group and 12 cases (36%) in HSCT group. Conventional testing detected pathogenic organisms in 6 cases (6%) of non-HSCT patients and 4 cases (12%) of HSCT patients which were negative by mNGS method. Pathogens identified by both mNGS and conventional testing were 27 (29%) cases in the non-HSCT group and 17 (52%) cases in the HSCT group. Pathogenic organisms identified by combining mNGS, the conventional testing results and clinical signs and symptoms included 16 (17%) cases in the non-HSCT group (**Figure 5A**). **Figure 5B** shows clinically determined pathogens detected only by mNGS rather than conventional methods in patients with hematological diseases, and 25 pathogens cannot be detected by conventional detection.

The clinical impact of mNGS in hematological patients was evaluated. mNGS had an overall beneficial impact on treatment in 61.70% of the non-HSCT patients and 75.8% of the HSCT patients. The beneficial impacts included initiation of antibiotic therapy (n=33 in non-HSCT, n=17 in HSCT), treatment de-escalation (n=10 in non-HSCT, n=2 in HSCT) and upgrading of anti-infection drugs (n=15 in non-HSCT, n=16 in HSCT). mNGS also identified pathogenic microorganisms in 18 cases (19%) in the non-HSCT group and 3 cases (9%) in the HSCT group. However, mNGS had no beneficial impact on treatment in 18 cases (19%) in the non-HSCT group and 5 cases (15%) in the HSCT group, because it could not direct the treatment or diagnosis of diseases (**Figure 5C**).

## Discussion

Hematological diseases, especially hematological malignancies, require intensive treatment with chemotherapy, hormones and immunotherapeutic and molecular targeted agents (Maschmeyer et al., 2019). Refractory or relapsed leukemia/lymphoma/multiple myeloma patients after multiple chemotherapy regimens usually undergo HSCT, so these patients are more prone to the development of bloodstream infections due to immune reconstruction, the use of immunosuppressants and other factors that damage the overall immune system. Opportunistic infections that do not cause disease in people with normal immunity may have serious clinical consequences in patients with hematological diseases and post-transplantation patients. As this article notes that infection is common complication and one of the main causes of death in patients with hematological diseases (Hardak et al., 2016). Consequently, it is very important to find pathogenic microorganisms in time. However, standard testing fails to identify pathogens in most patients with hematological disease. Due to the lack of specific diagnostic basis for fungal, viral and Mycobacterium tuberculosis infections, infections may be classified as bacterial related infections. This also reflects that the traditional detection methods cannot comprehensively detect all possible pathogens of hematological diseases, and it is difficult to understand the characteristics of infection spectrum in the real-world.

One study showed that Gram-positive cocci were the most commonly isolated (75.8%), while Gram-negative bacilli infections occurred in 12.1% of patients undergoing chemotherapy for acute myeloid leukemia (Madani, 2000). However, another study performed by Rima Moghnieh et al. showed that 57.3% of the 75 bacteremic cases were due to Gram-negative organisms, and the remaining 42.7% were Gram-positive in febrile neutropenia adult cancer patients. *Escherichia coli* and *Klebsiella pneumonia* were the most prevalent Gram-negative organisms, representing 22.7% and 13.3% of the total cases, respectively (Moghnieh et al., 2015). However, in our study, it showed that the proportion of Gram-negative bacilli was higher than that of Gram-positive cocci (28% *vs*. 10%). We speculated that the reason for inconsistent results in different studies might be related to the different bacterial spectra in different regions, hospitals and different inclusion conditions of the patients. In our study for non-HSCT patients, the proportion of Gram-negative bacilli was higher than that of Gram-positive cocci (33% *vs*. 7%), while Gram-positive cocci were predominant in HSCT patients (Gram-negative bacilli *vs*. Gram-positive cocci, 13% *vs*. 24%). We hypothesize that the cause of the large difference in pathogenic bacteria between HSCT and non-HSCT patients is that HSCT recipients undergoing intensive myelosuppressive, immunosuppressive treatment and immune reconstruction are at high risk for severe, life-threatening bacterial infections. Our study showed that *Klebsiella pneumonia* accounted for the highest proportion, followed by *Pseudomonas aeruginosa* and *Acinetobacter baumannii*. *Stenotrophomonas maltophilia* accounted for the largest number of Gram-negative bacilli in HSCT patients, followed by *Enterobacter cloacae* complex. However, other studies suggested that *Escherichia coli* was the most common Gram-negative bacillus (Moghnieh et al., 2015). There were significant differences between the above studies. The reason may be that the patients included in other studies were infected due to neutropenia after HSCT, while the patients in our study had different infection conditions.

Hematological patients, especially HSCT recipients, are immunosuppressed and have received multiple courses of antibiotics in the past, and infections are more likely to occur in these patients with poor immunity. Vancomycin-resistant Enterococcus has increasingly become a major nosocomial pathogen worldwide since its discovery in England and France in 1986 (O'Driscoll and Crank, 2015). Therefore, the possibility of vancomycin-resistant *Enterococcus faecium* should be considered during clinical treatment for patients with poor immunity if an empiric application of vancomycin is ineffective. Staphylococcus is the most common pyogenic coccus and is an important source of nosocomial cross-infection. Most staphylococci are nonpathogenic bacteria. When the patients are immunocompromised, some of the nonpathogenic Staphylococcus can proliferate and cause symptoms of infection. One study suggested that methicillin-resistant coagulase negative staphylococci (MRCNS) were the most common Gram-positive bacteria, representing 66% of Gram-positive bacteremias and 28% of total bacteremias in febrile neutropenia adult cancer patients (Moghnieh et al., 2015). In our study, it showed that *Enterococcus faecium* made up a relatively high proportion followed by *Staphylococcus epidermidis* of the Gram-positive cocci in non-HSCT patients. Another study showed the incidence of *Streptococcus pneumoniae* in HSCT recipients was 40.4 per 100 000 patients per year, which is 30-fold higher than that in patients without HSCT (van Veen et al., 2016), which was consistent with our research. The differences in the above results may be due to the different underlying diseases and antibiotics used by the patients. Therefore, when patients with hematologic diseases show symptoms of infection and staphylococci are detected, it was still necessary to carefully determine whether it is a pathogen based on the clinical conditions.

High doses of steroids, irradiation, and the application of immunosuppressive agents are all risk factors for CMV activation (Hebart et al., 1997). During the post-transplant period, CMV reactivation occurs in up to 41% of patients, and CMV infection is an important cause of death (Marchesi et al., 2018). Our study showed that the incidence of CMV-activated infection was the highest among the viral infections. A meta-analysis showed that CMV infection was significantly associated with an increased risk of overall mortality (OM) and non-relapse mortality (NRM) after allo-HSCT. However, preemptive antiviral therapy does not benefit patients; in contrast, it results in a twofold increased risk of OM and NRM (Giménez et al., 2019). Therefore, the long-term application of anti-CMV drugs needs to be evaluated based on the specific situation of each patient. EBV, an identified oncogenic virus, causes more than 7 different types of malignancies, usually in immunocompromised individuals. Furthermore, some individuals with primary immunodeficiencies exhibit extreme susceptibility to EBV-induced diseases, such as severe and often fatal infectious mononucleosis, hemophagocytic lymphohistiocytosis, lymphoproliferative disease, and/or EBV+ B-cell lymphoma (Tangye and Latour, 2020). EBV activation is a common complication after allogeneic hematopoietic stem cell transplantation (allo-HSCT). EBV-related posttransplantation lymphoproliferative disorders (EBV-PTLDs) are rare but potentially fatal complications associated with T-cell depletion of grafts, HLA mismatch, severe graft-versus-host disease (GVHD) (Liu et al., 2018). Our mNGS results revealed that viruses were common pathogenic microorganisms in both non-HSCT (44%) and HSCT (45%) patients. The serological positive detection rate of CMV was the highest, followed by EBV. Therefore, the possibility of CMV and EBV infection should be first considered if fever occurs in hematological patients with or without transplant. The *Torque teno* virus (TTV), a highly prevalent nonpathogenic anellovirus, is a potential novel candidate for monitoring immunosuppression (Studenic et al., 2021). TTV is associated with many autoimmune diseases, such as idiopathic hepatitis and systemic lupus erythematosus. The incidence of autoimmune diseases after HSCT is between 2.1% and 5.5% (Daikeler et al., 2013; Faraci et al., 2014), and TTV infection may be an important contributing factor (Maximova et al., 2015). Our study showed that TTV was detected in non-HSCT patients, which suggested attention should be paid to the relationship between TTV infection and autoimmune disease in the treatment of hematological patients, and relevant examinations should be conducted.

With the application of immunosuppressive drugs, the morbidity due to fungal infection has gradually increased. A minority of these fungal infections cause life-threatening infections (Strickland and Shi, 2021). Köhler et al. showed that common fungi include *Cryptococcus neoformans*, *Aspergillus fumigatus* and *Candida albicans* (Köhler et al., 2014). Mucormycosis, which is difficult to diagnose, is an angioinvasive fungal infection with high morbidity and mortality. Diagnosis is often delayed, and the disease tends to progress rapidly. Patients with hematological malignancies or transplantation are more vulnerable to mucormycosis. The increased detection rate of mucormycosis may be due to the application of immunosuppressants and modern diagnostic tools identifying previously uncommon genera/species. Corticosteroids and other immunosuppressive agents are risk factors for mucormycosis (Cornely et al., 2019). In our study, the detection rate of fungi in nontransplant patients (13%) was higher than that in transplant patients (8%). However, the detection rate of Mucor was the highest, followed by Candida and Aspergillus. The reason for the high incidence of mucormycosis in our study may be that the enrolled patients were patients with hematological malignancies or transplantation, and mNGS could detect this pathogen early.

Immunosuppressed patients were more prone to mixed infections (Dropulic and Lederman, 2016). In our study, 76.7% patients suffered malignant hematologic diseases and 23.3% patients with non-malignant hematologic diseases had immunosuppression or used immunosuppressants. Our study showed patients with mixed infection accounted for 67.00%. Among them, the mixed infection of bacteria and virus (25.99%) was the most common, which indicated that mNGS had certain advantages in detecting mixed infection of pathogenic microorganisms.

Our study showed the pathogen detection rate of mNGS is higher than that of traditional detection methods, such as PCR and blood culture. The proportion of pathogens detected by mNGS was 85.82% and by conventional testing was 20.47%. The detection rate of mNGS for bacteria, fungi and viruses was higher than that of conventional testing, and even special pathogens, such as mycoplasma, *Mycobacterium tuberculosis* and Leishmania, which are difficult to cultivate, were detected. In the published literature (EORTC International Antimicrobial Therapy Cooperative Group, 1990; Madani, 2000; Collin et al., 2001; Moghnieh et al., 2015; Al-Otaibi et al., 2016; van Veen et al., 2016; Marchesi et al., 2018; Alonso-Álvarez et al., 2021), the majority of etiological analyses of hematological diseases have focused on bacterial and viral infections, while the analysis of fungal infections and special pathogen infections is relatively rare. In addition to the rarity of these pathogen infections, the detection technology is still not perfect, and the infection may be mistakenly attributed to bacterial infection. We hypothesize that the reasons for these phenomena are not only that the pathogenicity of these pathogens is low but also that identification of the etiological agents remains challenging because only a small proportion of pathogens are identifiable by the current diagnostic methods. Hence, mNGS has become an attractive strategy and is an objective, unbiased, and comprehensive method for the detection and taxonomic characterization of microorganisms (Li et al., 2021).

The application of mNGS in the real world was evaluated in this study. In the non-HSCT group, 48% of pathogens were identified by mNGS only, and it was 36% in the HSCT group. Conventional testing detected pathogenic organisms in 6% of non-HSCT patients and 12% of HSCT patients. Pathogens identified by both mNGS and conventional testing were 29% of cases in the non-HSCT group and 52% of cases in the HSCT group. Pathogenic organisms identified by combining mNGS, the conventional testing results and clinical signs and symptoms were 17% of cases in the non-HSCT group. There were 25 pathogens, such as Leishmania, which could only be detected by mNGS. Therefore, mNGS plays an important role in identifying pathogens for a clinical diagnosis, as well as having an overall beneficial impact on treatment, such as the rapid initiation of appropriate antibiotic therapy, treatment de-escalation and upgradation of anti-infection drugs. Meanwhile, mNGS also identified pathogenic microorganisms and directed the application of antibiotics. Consistent with the results of a previous study (Zhan et al., 2021), our study showed that mNGS has a significant role in guiding physicians to make personalized treatment plans for patients.

Although the success of mNGS in improving the diagnosis, treatment, and tracking of infectious diseases was confirmed compared with other traditional methods, the limitations of mNGS are worthy of our attention. First, the human host background and extraneous sources of nucleic acids may influence the testing results (Marotz et al., 2018). Second, infective pathogens cannot be differentiated from colonizing pathogens. Because the human microbiome is complex and certain parts of the human body are naturally colonized by many bacteria, fungi, and viruses, primarily within the respiratory and gastrointestinal tracts, skin, and vagina, while most areas within healthy individuals are physiologically sterile, with microorganisms present only under certain conditions. Interpretation of mNGS report is therefore quite difficult and requires experience in clinical, laboratory and microbiology research. Therefore, in determining the clinical significance of the detected microbe, the whole clinical situation must in all cases be taken into account (Li et al., 2021). Third, the high cost of mNGS detracts from the advantages of its high sensitivity and a short detection cycle, which limits the clinical application of mNGS (Li et al., 2021). Consequently, this technology should be further optimized, which could make mNGS more accurate and cost-effective and improve its universality.

As our study is a retrospective study, we just analyzed the pathogens of HSCT and non-HSCT group in hematological diseases and we just made summary and analysis of our existing data. However, since hematological diseases included many subtypes, the sample size of each subtype was relatively small, which is the shortcoming of our paper. Continuous studies will focus on the presence of specific pathogen associated with certain type of malignancies.

## Conclusions

Our retrospective and monocentric study clinically showed mNGS has high clinical recognition rate and sensitivity in pathogen detection and provides a basis for guiding the anti-infective treatment in hematological diseases with symptoms such as fever. Simultaneously, the detection rate of mixed infections by mNGS was high. But mNGS still could not replace the traditional detection methods. mNGS of peripheral blood can be used as an important supplementary detection method when traditional tests are negative, or specimens are difficult to obtain. mNGS is rapidly transforming from the laboratory to the clinic with its advantages, but the timing of its clinical use needs to be further explored.

## Data availability statement

The original contributions presented in the study are included in the article/supplementary material. Further inquiries can be directed to the corresponding authors.

## Ethics statement

The studies involving human participants were reviewed and approved by Ethics Committee of the First Affiliated Hospital of Zhengzhou University.

## Author contributions

JY and ZJ conceived and designed the experiments. XZ and FW collected clinical samples and data. XZ wrote the manuscript. All authors contributed to the article and approved the submitted version.
